# Digit revascularization in patient with congenital monodactyly: expanding indications for salvage

**DOI:** 10.1093/jscr/rjag905

**Published:** 2026-10-07

**Authors:** Alisa O Girard, Shruthi Deivasigamani, Srivalli S Valluri, Michael J Franco

**Affiliations:** Division of Plastic and Reconstructive Surgery, Cooper University Hospital, 1 Cooper Plaza, Camden, NJ 08103, United States; Cooper Medical School of Rowan University, 401 Broadway, Camden, NJ 08103, United States; Division of Plastic and Reconstructive Surgery, Cooper University Hospital, 1 Cooper Plaza, Camden, NJ 08103, United States; Cooper Medical School of Rowan University, 401 Broadway, Camden, NJ 08103, United States; Cooper Medical School of Rowan University, 401 Broadway, Camden, NJ 08103, United States; Division of Plastic and Reconstructive Surgery, Cooper University Hospital, 1 Cooper Plaza, Camden, NJ 08103, United States; Cooper Medical School of Rowan University, 401 Broadway, Camden, NJ 08103, United States

**Keywords:** digit replantation, revascularization, monodactyly, congenital hand difference, microsurgery, dog bite

## Abstract

Management of partial and complete digit amputations is guided by injury characteristics, patient comorbidities, and functional prognosis; however, congenital hand differences and disability remain underexplored in current literature. We present a 47-year-old congenitally blind patient with tetramelic monodactyly who sustained a near-complete zone II amputation of his only left digit following a dog bite, with associated metacarpal head destruction. Despite relative contraindication conferred by the mechanism of injury, the patient had profound reliance on his digit for independence; as such, emergent revascularization was performed via metacarpophalangeal joint (MCPJ) arthrodesis and repair of a single digital artery and nerve. Venous outflow was facilitated by topical heparin-soaked pledgets and acetylsalicylic acid. At five months, the patient demonstrated meaningful MCPJ motion, sensory restoration, and return to activities of daily living. This case highlights the critical role of patient-centered social factors and reconstructive principles in surgical decision-making when established prognostic frameworks offer limited guidance.

## Introduction

Nearly 500 000 individuals sustain digit amputations in the USA annually [1]; however, single-digit replantation is fairly uncommon [2]. General indications and contraindications for digit replantation align with projected digit survival, function, and quality of life [3]. While these factors have largely reached expert consensus, surgical decision-making requires careful consideration of individual patient factors. Current literature underexplores circumstances of aberrant anatomy which may challenge surgical salvage and functional prognosis, as seen with congenital hand differences. A case of near-complete amputation of a congenitally anomalous digit is presented herein for consideration among current perspectives in digit salvage.

## Case report

A 47-year-old male with congenital blindness and tetramelic monodactyly presented to the hospital after a dog bite, resulting in a near-complete amputation of his only left digit and a crushed metacarpal head (Figs 1 and 2). The distal interphalangeal joint was congenitally fused, and the fingertip was pale and cool without distal flow on pinprick or Doppler ultrasound. Prior documentation or vascular studies were not available, and he had no history of nicotine use, diabetes mellitus, or peripheral vascular disease. The patient reported reliance on his digits for activities of daily living with excellent family support at home. Thus, emergent revascularization was attempted.

**Figure 1:**
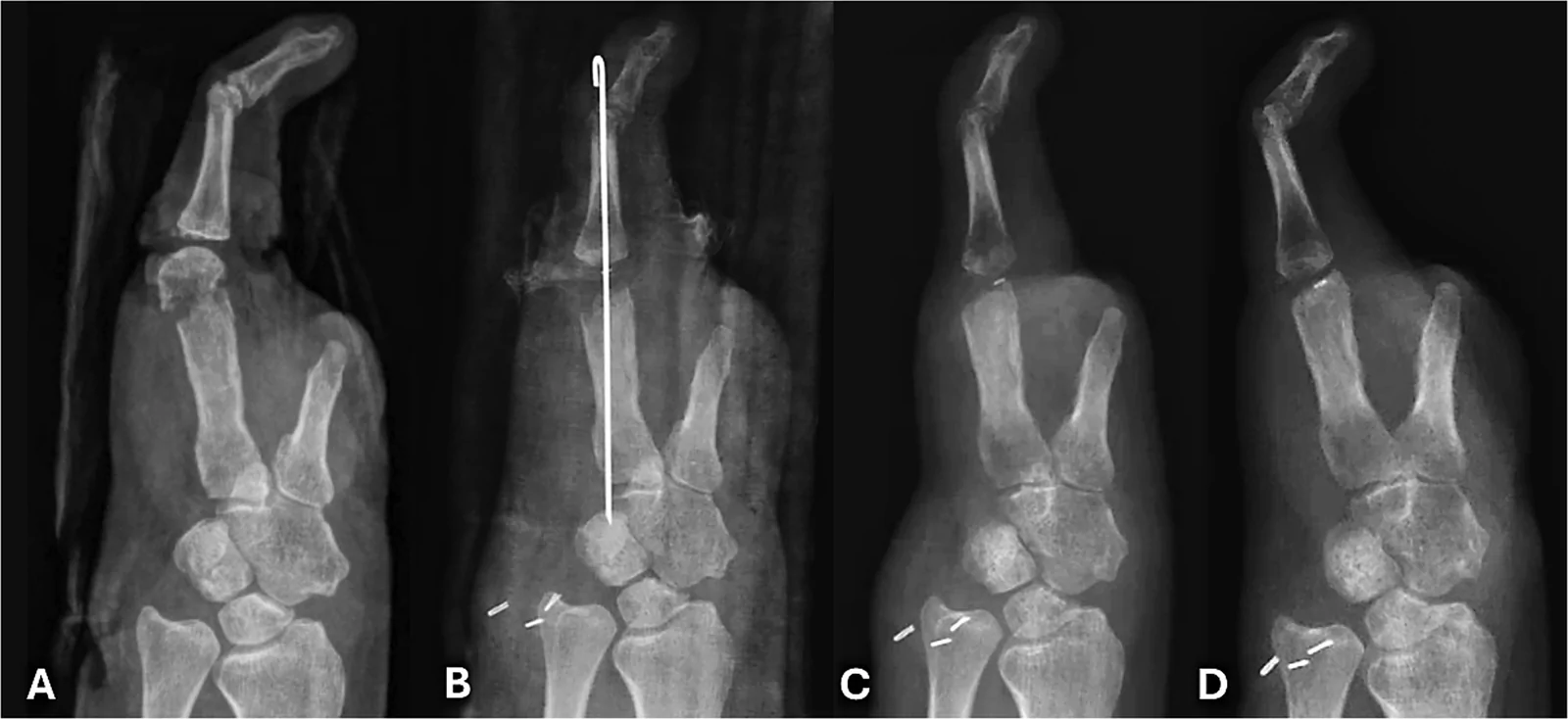
Radiograph of left hand on day of injury, demonstrating crush injury to metacarpal head and soft tissue at the base of the digit (A), day of pin removal, POD24, with a Kirschner wire spanning the metacarpal and proximal phalanx (B), POD38 (C), and POD80 with appropriate interval healing (D).

**Figure 2:**
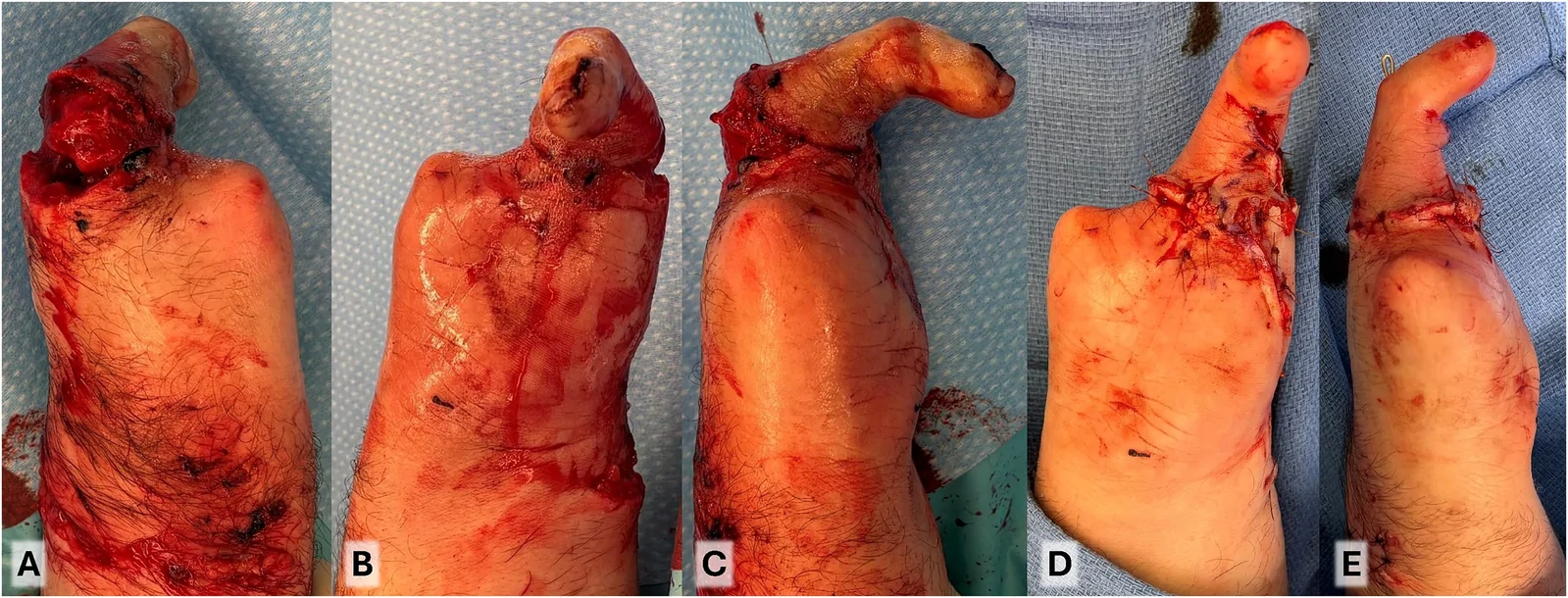
Preoperative photographs demonstrating near-complete amputation through the metacarpophalangeal joint with exposed bone and loss of the extensor mechanism (A–C) and postoperative photographs demonstrating a percutaneous Kirschner wire, loose closure of soft tissues, and a well-perfused digit (D and E).

Following copious irrigation under tourniquet, intact structures were identified: the flexor tendon apparatus and a narrow bridge of soft tissue containing superficial veins. The unsalvageable metacarpal head was excised, and arthrodesis of the remnant metacarpophalangeal joint (MCPJ) was performed with a 1.1 mm Kirschner wire. With structures stabilized and reapproximated, the extensor tendon was repaired next. The radial digital artery and bilateral veins were deemed nonviable; however, the ulnar digital artery was amenable to tensionless primary repair. After completion of the anastomosis, capillary refill returned with bright red bleeding on pinprick. Following neurorrhaphy of the ulnar digital nerve, the skin was loosely closed.

Heparin-soaked pledgets were applied to the nailbed hourly for 24 h to facilitate venous outflow. Strict hand elevation under a heating pad, oral acetylsalicylic acid (ASA 81 mg) daily, and intravenous ampicillin-sulbactam were employed. The patient was discharged on postoperative day (POD) 1 on amoxicillin–clavulanate (500–125 mg twice daily, 5 days) and ASA 81 mg. The K-wire was removed on POD24, and aggressive occupational therapy was initiated. At 5 months, MCPJ motion was modestly restored, sensation returned without pain, and he resumed digit use for daily activities.

## Discussion

This case depicts successful digit revascularization in the setting of a contaminated avulsion-crush injury proximal to the flexor digitorum superficialis insertion (zone II, “no man’s land”) with associated joint destruction. The potential for microsurgical repair and functional restoration of congenitally aberrant structures was unknown. In the absence of digit-threatening comorbidities (e.g. smoking) [3–6] or life-threatening psychiatric or medical conditions [4], the decision to attempt salvage was largely driven by social factors.

### Preoperative evaluation

The patient was congenitally blind with one digit on each hand bearing baseline deficits in range of motion; however, he was well adapted to using his digits for activities of daily living. He expressed that loss of the finger would significantly diminish his independence and quality of life. In a survey of plastic surgeons, most would replant a single finger amputation through zone II if the patient were a musician (97.1%), unemployed (80.0%), or a laborer (63.8%) [2]. Prior studies also discuss ethnic and cultural factors in decision-making; however, disabilities are not considered [2, 7]. Inability to care for a digit postreplantation is a relative contraindication to salvage attempt [3]. In this case, the patient’s strong home support and motivation to rehabilitate postoperatively optimized salvage outcomes.

A thorough discussion of surgical risks and prognostic indicators is critical to establish appropriate outcomes and care expectations, particularly with respect to the patient’s baseline activity. Salvage rates are lower in the setting of crush rather than sharp amputations given the extended zone of injury [8, 9]. Wide debridement of nonviable structures may delay or limit functional rehabilitation secondary to bone shortening, joint immobilization, tenuous tendon repair, and neurovascular reconstruction with grafts. Anticipation of suboptimal functional outcomes and patient dissatisfaction are commonly cited reasons to forego replantation [2].

### Surgical considerations

Perhaps the greatest predictor of salvage in this case was partial rather than complete amputation of the digit. Prior literature reports greater survival rates in digit revascularization (80%) than replantation (48%); however, the heterogeneity of remaining intact structures should be considered [8].

Intact venous tissue confers digit survival benefit, thereby substantiating careful preservation of viable soft tissue bridges [10]. As this was a contaminated case, crushed structures required aggressive debridement and thorough irrigation [11].

Surgical digit shortening enabled tensionless primary artery repair without a graft [6, 9]. At the proximal level of injury, anastomosis of the larger caliber artery was also more feasible, enabling reliable digit reperfusion on a single vessel [8].

Current evidence varies with respect to ischemia time windows for digit reperfusion; however, 12 h of warm ischemia and 24 h of cold ischemia are the most frequently reported prognostic cutoffs [6, 8, 12, 13]. Perfusion was restored 9 h following injury—well within these windows.

### Perioperative care

The risks and benefits of systemic anticoagulation remain controversial; however, prior studies demonstrated improved salvage rates with topical heparin and ASA, as used in this case [10, 14]. Topical heparin diminishes risk of venous congestion. Postoperative digit warming with heating blankets is also routinely employed at this hospital to optimize perfusion [14].

Although patient, injury, and technical considerations for digit replantation have been broadly investigated, management is fundamentally guided by surgeon experience [8]. Microsurgeons may be more willing to attempt replantation and salvage success rates are concordantly higher at high-volume (three or more annually) replantation centers [2, 15]. This surgical case was performed at a high-volume center by an experienced hand/microsurgeon with clinical judgment informed by empirical, anecdotal, and heuristic evidence. Thus, unique social and anatomical factors in this case were heavily weighed against poor prognostic indicators within the limits of consensus guidelines. While the current literature offers little guidance on replantation in the setting of congenital hand differences, this case underscores the importance of patient autonomy and adherence to reconstructive principles to optimize outcomes in ethically and technically challenging circumstances.

## Data Availability

Available upon reasonable request.
